# Need for integration of hepatitis C (HCV) services in community-based settings for people who inject drugs: results from a global values and preferences survey

**DOI:** 10.1186/s12954-023-00743-8

**Published:** 2023-02-09

**Authors:** M. Di Ciaccio, V. Villes, C. Perfect, J. L. El Kaim, M. Donatelli, C. James, P. Easterbrook, R. M. Delabre

**Affiliations:** 1 Community-Based Research Laboratory, Coalition PLUS, Pantin, France; 2 Advocacy Department, Coalition PLUS, Pantin, France; 3 World Hepatitis Alliance, London, UK; 4 Department of Global HIV, Hepatitis and STI Programmes, World Health Organization, Geneva, Switzerland

**Keywords:** People who inject drugs, HCV services, Values and preferences, Decentralisation, Integration, Task shifting

## Abstract

**Background:**

To inform the development of updated World Health Organization (WHO) guidelines on simplified service delivery for HCV infection, a global survey was undertaken among people affected or infected by HCV. The objective of this analysis is to identify specific needs and preferences among people who inject drugs.

**Methods:**

A multi-country, anonymous, self-administered online survey conducted in 2021 was developed by Coalition PLUS and the World Hepatitis Alliance in partnership with the WHO. Preferences for test and treat locations and simplifying HCV care were collected among people affected or infected by HCV. Chi-square tests were used to compare respondents who identified with current or former injection drug users through identification with key population to other respondents who did not identify with this key population.

**Results:**

Among 202 respondents, 62 (30.7%) identified with current/former injection drug users. Compared to other respondents, they were: older [median (IQR): 48 (36–57) vs. 39 (31–51) years, *p* = 0.003]; more likely to have been tested for HCV (90.2% vs. 64.3%, *p* = 0.001); more likely to prefer testing in a community-based centre (CBC) (55.4% vs. 33.3%, *p* = 0.005); or in a support centres for people who use drugs (SCPUD)(50.0% vs. 9.8%, *p* < 0.001). The most important considerations regarding testing locations among people identified with current/former injection drug users (compared to the other respondents) were: non-judgemental atmosphere (*p* < 0.001), anonymity (*p* = 0.018) and community worker (CW) presence (*p* < 0.001). People identified with current/former injection drug users were more likely to prefer to receive HCV treatment in a CBC (63.0% vs. 44.8%, *p* = 0.028) or in a SCPUD (46.3% vs. 9.5%, *p* < 0.001), compared to the other respondents. The most important considerations regarding treatment locations among people identified with current/former injection drug users were the non-stigmatising/non-judgemental approach at the site (*p* < 0.001) and the presence of community-friendly medical personnel or CW (*p* = 0.016 and 0.002), compared to the other respondents.

**Conclusion:**

The preferences of people identified with current/former injection drug users indicated specific needs concerning HCV services. Integration of HCV services in community-based risk reduction centres may be an important element in the development of adapted services to increase uptake and retention in HCV care among this population.

## Introduction

Hepatitis C virus (HCV) is a major cause of chronic liver disease with a global estimation of 58 million people living with chronic HCV infection in 2019 [1]. People who inject drugs are highly exposed to HCV through certain injection practices such as sharing needles or other injection equipment [2–5]. Globally, 14 million people who inject drugs were estimated at high risk of HCV infection due to their injection practices [6], and HCV prevalence among people who inject drugs was estimated at 52.3% (95% UI 42.4–62.1) in 2015 [7]. Therefore, HCV prevention is mainly based on the risk reduction strategies associated with injection behaviours. Indeed, it has been estimated that 43% of new HCV infections from 2018 to 2030 could be prevented if transmission risk due to injection drug use was eliminated [8]. Harm reduction services (HRS) aim to reduce these risks; however, a global systematic review of interventions to prevent HCV infection among people who inject drugs showed a lack of coverage in HCV prevention services (including needle and syringe programmes and opioid agonist treatment), mainly in regions outside Europe [9]. Moreover, access to HRS may also be impeded by marginalisation and criminal sanctions against people who inject drugs [10, 11]. Marginalisation and stigma may lead to reduced awareness and uptake of HRS [12–14]. Using a multidimensional drug use stigma scale, a large cross-sectional study in 12 cities in India showed that various forms of stigma were significantly associated with HCV infection [15].

Although an effective HCV treatment is now available, access to treatment has suffered from the same barriers as HRS (lack of coverage, effects of marginalisation and stigma). HCV services (treatment or prevention) were not well adapted to people who inject drugs [16, 17]. Other studies identified additional barriers to HCV treatment such as competing priorities and limited healthcare access [18–21]. Finally, the HCV care pathway has also been characterised by long delays between each step, administrative hurdles, lack of proximity of clinics and long waits at clinics, high out-of-pocket direct and indirect costs [22].

The lack of HRS coverage, linkage to care and access to HCV treatment among people who inject drugs are factors which may reduce the benefits of HCV treatment as prevention, a key strategy to eliminating HCV transmission [23]. In this context, there is a growing interest in providing an integrated model of HCV care for people who inject drugs to facilitate progression through the HCV continuum of care [24]. Community-based HCV services, which engage all stakeholders affected by HCV, could be well placed to identify and respond to the specific needs of people who inject drugs and address stigma issues [17, 25]. Studies have already shown that community-based HCV (self) testing was highly acceptable [26, 27], and one of most preferred ways for testing among people who inject drugs, due to stigma encountered in other healthcare settings [28, 29]. Furthermore, studies implementing simplified HCV testing and treatment initiation in community-based settings, and with different levels of community-based engagement throughout the continuum of HCV care [30–32], have provided encouraging evidence to pursue this approach. Many other studies aimed to increase uptake of HCV prevention and care among people who inject drugs through decentralisation of HCV services. Indeed, a recent WHO systematic review and meta-analysis of 142 studies, of which 14% were in low and middle-income countries, examined the effectiveness of key simplified service delivery interventions—decentralisation, integration and task sharing to non-specialists on outcomes across the HCV cascade of care in four distinct populations, including people who inject drugs and persons in prisons [33, 34]. The review showed that the impact of full decentralisation/integration of HCV testing and treatment was greatest among people who inject drugs and prisoners than in people living with HIV or among the general population.

The values and preferences of people who inject drugs are therefore essential for developing and/or adapting HCV service delivery to meet their specific needs. To inform key 2022 WHO guidelines updates on simplified service delivery of HCV testing and treatment [34], a survey was conducted to identify the values and preferences of people living with or affected by HCV, including people who inject drugs, regarding decentralisation, integration and task shifting of HCV care services.

The present study explores the specific preferences of people who identified current/former with injection drug users compared to other survey respondents.

## Methods

### Design and objectives

Data came from a cross-sectional, anonymous, self-administered online survey that aimed to collect information regarding the values and preferences of people identified as living with HCV, or people identified as likely to be exposed to or affected by HCV (such as people who, currently or formerly, inject drugs; people living with HIV; and prisoners) through identification with key population, on simplifying delivery of care and treatment for chronic HCV. The results of this survey were used alongside key evidence from a systematic review [33], as well as cost-effectiveness data to inform key updates to HCV testing and treatment recommendations in WHO guidelines [34].

### Survey population

This survey targeted participants who were aged 18 years or older and who were living with or affected by HCV. All participants were provided with information regarding the objectives of the survey. Study participants had to confirm their willingness to participate before starting the survey. No identifying information was collected for the respondents.

### Survey development and promotion

The survey was developed by Coalition PLUS, an international union of community-based organisations (16 member organisations and over 100 partner organisations present in 52 countries) involved in the fight against HIV and viral hepatitis, and the World Hepatitis Alliance, an international network of organisations (civil society and community organisations) working in over 100 countries toward the goal of eliminating hepatitis by 2030, in partnership with the WHO (The WHO invited Coalition PLUS, World Health Alliance and TAG to participate in the development and diffusion of the values and preferences survey). The survey was distributed via civil society email networks, social media and mailing lists. Information concerning preferences for simplifying HCV care, and test and treat locations, were collected. More details of the study design and promotion are available in the Web Annexe D of the updated WHO recommendations on HCV simplified service delivery [35] and will be published elsewhere [36].

### Data collection

The online survey was launched on 8 September 2021. Data were collected over 2 weeks, with the survey closing on 22 September 2021.

### Data collection

The questionnaire collected information including sociodemographic characteristics: age, gender (male, female, transgender, gender non-binary), living area (urban, semi-urban, rural) and highest level of education completed (tertiary, other) and previous testing and treatment for HCV (tested at least once, and among them, previously tested positive and then, positive tested with RNA PCR and among them, treated for HCV). Information about preferences regarding HCV testing and treatment locations was collected, for which respondents could indicate up to three choices among the 11 following for testing: hospital, primary care clinic, community-based organisation centre, sexual and reproductive health clinics, support centres for people who use drugs, laboratory, HIV Clinic; pharmacy, place of my choice using a self-test, no preference and other; and 9 for treatment: Hospital, HIV Clinic, support centres for people who use drugs, community-based organisation centre, sexual and reproductive health clinics, general practitioner’s cabinet, same site as testing site, no preference and other. Respondents also were asked to report the most important considerations regarding these locations (possibility to select up to three among the 10 following for testing: close to your home or workplace, service hours, waiting time, direct costs, indirect costs, presence of community/peer workers, presence of community-friendly medical personnel, non-judgemental atmosphere, anonymity and other; and 13 for treatment: close to your home or workplace, service hours, waiting time, direct costs, indirect costs, confidence in personnel, presence of community/peer workers, presence of community-friendly medical personnel, non-judgemental atmosphere, anonymity, no preference, none of these proposals and other).

### Statistical analysis

For the present analysis, two groups were used based on identification or not of current/former injection drug users through identification with key population. Chi-square or Fisher tests for categorical variables and Wilcoxon–Mann–Whitney tests for continuous variables were used to compare respondents who identified with current/former injection drug users to other respondents. Percentages and *p* values were used to compare the differences between groups regarding the preferences on testing and treatment services and considerations for these choices. We then identified the three most important within each group.

## Results

### Characteristics of the study population

Respondents came from a large number of countries within 6 WHO regions [35]. However, most respondents were from Nigeria (23%), the USA (20%), Australia (7%) and the UK (7%).

Among 202 respondents (Table 1), median age was 42, 56.5% were male, 40.5% were female, 2.0% were gender non-binary and 1.0% was transgender. More than two-thirds (68.8%) lived in an urban area (large city/near a large city) and 88.4% completed higher education level (college, university or vocational training).

**Table 1:** Description and comparison of socio-demographics characteristics and of previous testing and treatment for HCV among respondents identified with current/former injection drug users compared to other respondents, *N* = 202

	Other respondents *n* = 140 (69.3%)	Respondents identified with current/former injection drug users *n* = 62 (30.7%)	Total *N* = 202	*p* value
	*n* (%)	*n* (%)	N (%)	
*Age median [IQR] (n* = *189)*	39 [31–51]	48 [36–57]	42 [33–52]	**0.003**
*Gender (n* = *200)*				0.393
Male	76 (54.7)	37 (60.7)	113 (56.5)	
Female	60 (43.2)	21 (34.4)	81 (40.5)	
Transgender	1 (0.7)	1 (1.6)	2 (1.0)	
Gender non-binary	2 (1.4)	2 (3.3)	4 (2.0)	
*Living area (n* = *199)*				**0.022**
Urban area/large city/Near a large city	93 (67.4)	44 (72.1)	137 (68.8)	
Semi-urban area/small or medium city	33 (23.9)	6 (9.8)	39 (19.6)	
Rural area/Village	12 (8.7)	11 (18.0)	23 (11.6)	
*Highest level of education completed (n* = *198)*				0.379
Never went to school/primary/secondary (middle school/high school)	15 (10.8)	9 (15.3)	24 (12.1)	
Tertiary (college, university, or vocational training)	124 (89.2)	50 (84.7)	174 (87.9)	
*Previously tested for HCV (n* = *201)*				**0.001**
Yes	90 (64.3)	55 (90.2)	145 (72.1)	
No/I do not know	50 (35.7)	6 (9.8)	56 (27.9)	
*Previously tested positive for HCV (n* = *139)*^*a*^				** < 0.001**
*Yes*	19 (22.6)	38 (69.1)	57 (41.0)	
*No*	65 (77.4)	17 (30.9)	82 (59.0)	
*Previously tested positive with RNA PCR for HCV (n* = *57)*^*b*^				NA
Yes	18 (94.7)	36 (94.7)	54 (94.7)	
No/I do not know	1 (5.3)	2 (5.3)	3 (5.3)	
*Previous treatment for HCV (n* = *53)*^*c*^				0.153
Yes	18 (100.0)	30 (85.7)	48 (90.6)	
No	0 (0.0)	5 (14.3)	5 (9.4)	

Missing values were excluded of this comparison/*P*-values in bold are significant

^a^Among those previously tested for HCV

^b^Among those previously tested positive for HCV

^c^Among those previously tested positive with RNA HCV for HCV

**Fig. 1:**
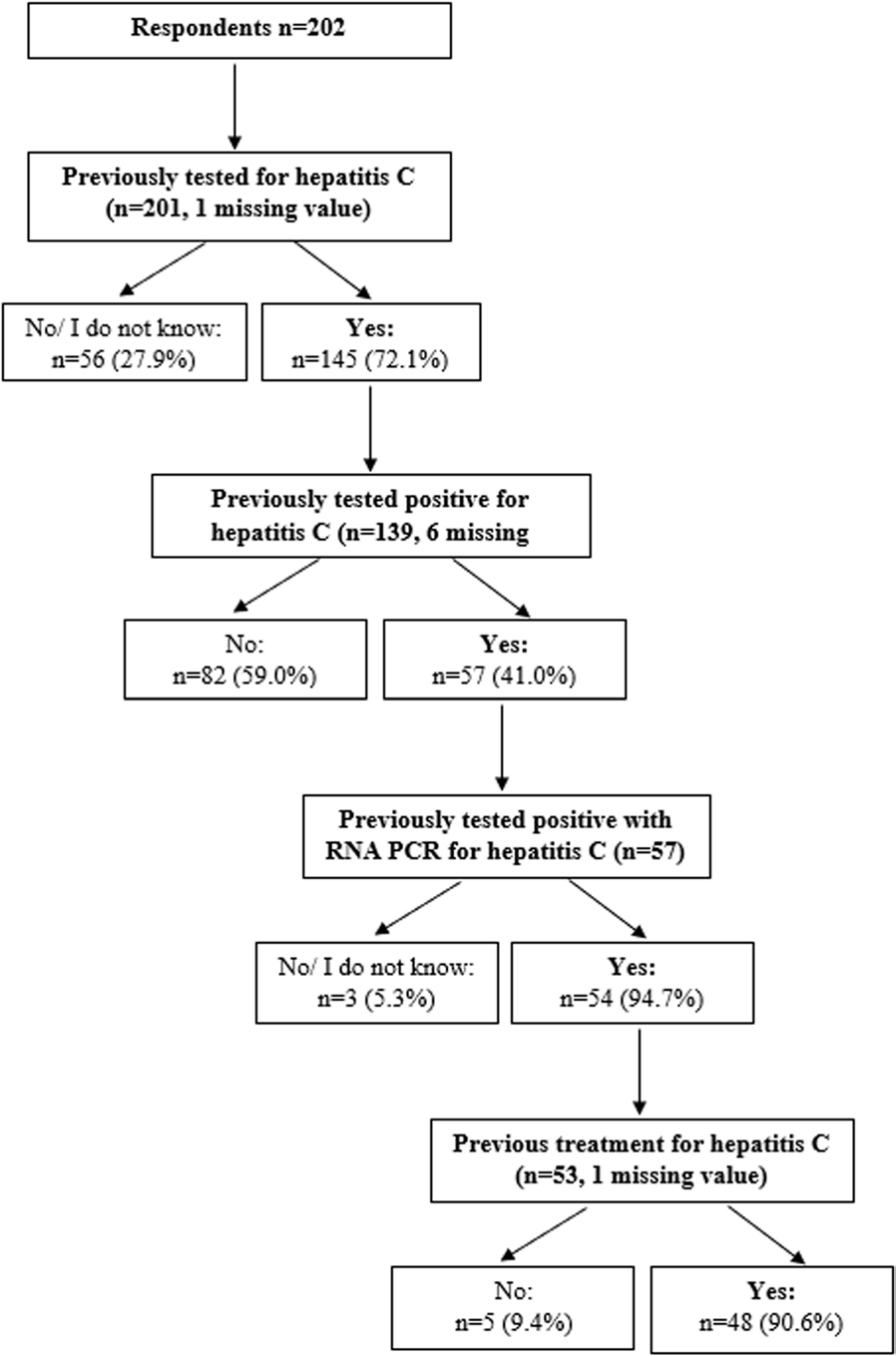
Description of testing and treatment for HCV (*n* = 202)

Almost three-quarters (*n* = 145, 72.1%) had been previously tested at least once for HCV and among them, 39.3% (*n* = 57) had been previously tested positive of which 94.7% (*n* = 54) tested positive with RNA PCR. Among them (*n* = 54), 88.9% (*n* = 48) have already been treated for HCV (Fig. 1). Concerning HCV testing locations, the three places most preferred by participants were in a community-based centre (CBC) (40.2%), followed by “place of my choice, using a self-test” (34.1%) and in a hospital (33.0%). The three main considerations regarding HCV testing location were a place “close to your home or workplace” (54.4%), direct costs (37.2%) and a place with a non-judgmental atmosphere (35.6%). Concerning HCV treatment locations, the three places where participants would like to receive the treatment were in “the same site as testing site” (54.7%) followed by in a CBC (50.6%) and in a hospital (39.4%). The three main considerations regarding HCV treatment location were a place at “proximity to my home or workplace” (56.5%), direct cost (39.4%) and a place with a non-stigmatising/non-judgmental approach (38.8%).

### Comparison of respondents who identified with current/former injection drug users to other respondents

Among the 202 respondents, 62 (30.7%) identified with current/former injection drug users. Compared to respondents who did not identify with current/former injection drug users, respondents who identified with current/former injection drug users were older (median: 48 vs. 39 years, *p* = 0.003; Table 1) and they more often lived in an urban area (large city/near a large city) (72.1% vs. 67.4%, *p* = 0.022). Respondents who identified with current/former injection drug users were more likely to have already been tested for HCV (90.2% vs. 64.3%, *p* = 0.001). Among those previously tested for HCV, respondents who identified with current/former injection drug users also were more likely to have already been tested positive for HCV (69.1% vs. 22.6%, *p* < 0.001). Among those previously tested positive with RNA PCR for HCV, respondents who identified with current/former injection drug users were less likely to have previous treatment for HCV (85.7% vs. 100.0%, *p* = 0.153).

**Fig. 2:**
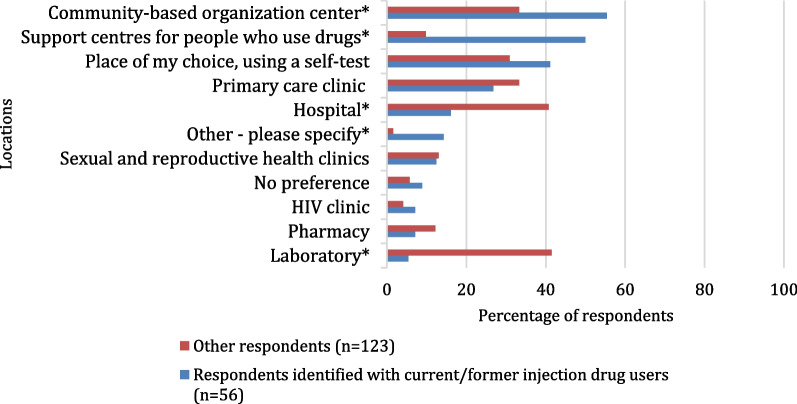
Preferences regarding HCV testing locations^1^ among participants identified with current/former injection drug users and other participants (*n* = 179). Possibility to select up to three locations. *p value < 0.05. ^1^Locations choices which did not allow a comparison between groups were excluded

**Fig. 3:**
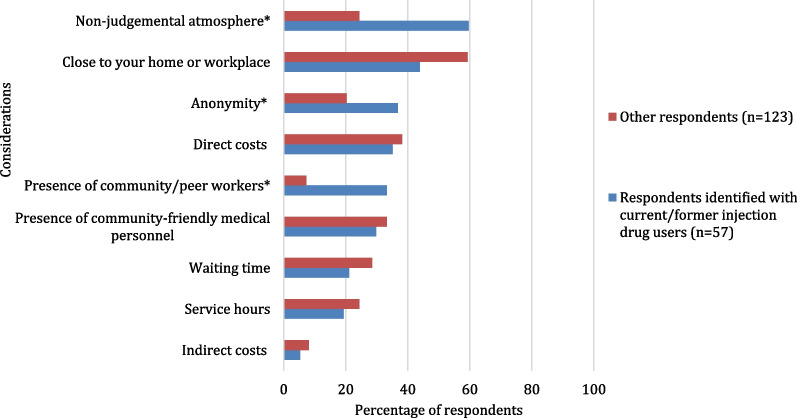
Most important considerations^1^ regarding HCV testing locations among participants identified with current/former injection drug users and other participants (*n* = 180). Possibility to select up to three considerations. *p value < 0.05. ^1^Considerations choices which did not allow a comparison between groups were excluded

Concerning the preferences for HCV testing location (Fig. 2), people who identified with current/former injection drug users had lower preference for testing in a hospital (16.1% vs. 40.7%, *p* = 0.001) or in a laboratory (5.4% vs. 41.5%, *p* < 0.001) compared to other respondents. In contrast, the preference to be tested in a community-based centre (CBC) (55.4% vs. 33.3%, *p* = 0.005) or in a support centre for people who use drugs (SCPUD) (50.0% vs. 9.8%, *p* < 0.001) was higher among respondents who identified with current/former injection drug users. The most important considerations regarding preferences for testing locations of respondents who identified with current/former injection drug users (Fig. 3) were the non-judgemental atmosphere (59.6% vs. 24.4%, *p* < 0.001), anonymity (36.8% vs. 20.3% *p* = 0.018) and community worker (CW) presence (33.3% vs. 7.3%, *p* < 0.001).

**Fig. 4:**
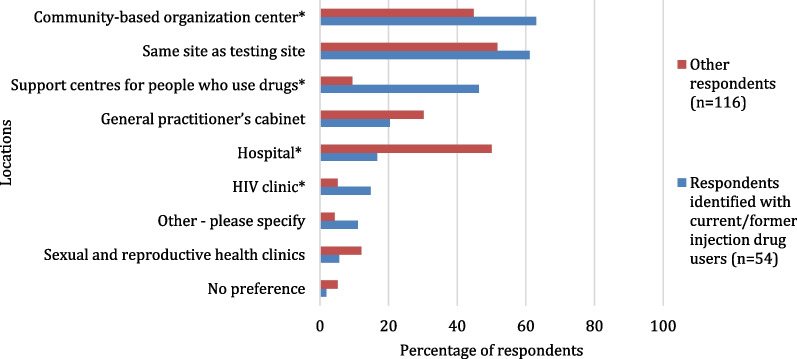
Preferences regarding HCV treatment locations^1^ among participants identified with current/former injection drug users and other participants (*n* = 170). Possibility to select up to three locations. *p value < 0.05. ^1^Locations choices which did not allow a comparison between groups were excluded

**Fig. 5:**
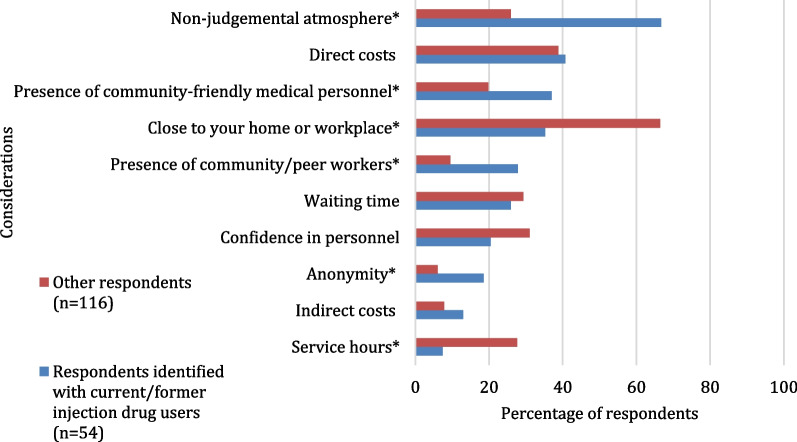
The most important considerations^1^ regarding HCV treatment locations among participants identified with current/former injection drug users and other participants (*n* = 170). Possibility to select up to three considerations. *p value < 0.05. ^1^Considerations choices which did not allow a comparison between groups were excluded

Regarding HCV treatment locations preferences (Fig. 4), respondents who identified with current/former injection drug users were less likely to prefer receiving HCV treatment in a hospital (16.7% vs. 50.0%, *p* < 0.001) and would like to receive HCV treatment in a CBC (63.0% vs. 44.8%, *p* = 0.028) or in a SCPUD or HIV clinic (46.3% vs. 9.5%, *p* < 0.001 and 14.8% vs. 5.2%, *p* = 0.033, respectively). The most important considerations regarding treatment locations (Fig. 5) of respondents who identified with current/former injection drug users were the non-stigmatising/non-judgemental approach (66.7% vs. 25.9%, *p* < 0.001), the presence of community-friendly medical personnel or CW (37.0% vs. 19.8%, *p* = 0.016 and 27.8% vs. 9.5%, *p* = 0.002, respectively) and anonymity (18.5% vs. 6.0%, *p* = 0.012). “Proximity to my home or workplace” and service hours were less important factors regarding HCV treatment services for respondents who identified with current/former injection drug users compared to other respondents (35.2% vs. 66.4%, *p* < 0.001 and 7.4% vs. 27.6%, *p* = 0.003, respectively).

## Discussion

Results of this international survey showed that respondents who identified with current/former injection drug users had specific needs concerning HCV services compared to other respondents. Respondents who identified with current/former injection drug users had a history of drug injection were more frequently interested in testing at CBC or support centres for people who use drugs, and they considered a non-judgmental approach, anonymity and CW presence as important components of HCV testing services. Similar results were observed for HCV treatment preferences in terms of location (CBC, support centres for people who use drugs, HIV clinic) and key components (non-stigmatising/non-judgemental approach, presence of community-friendly medical personnel or CW and anonymity). Practical and technical components, such as service hours, proximity to home or workplace of HCV services were considered significantly less important compared with respondents who did not have a history of injecting drugs.

These results highlight the need to diversify HCV service pathways to better reach people who inject drugs. Their preferences in terms of HCV services in this study support the integration of HCV services in community-based or community-friendly settings. These results could be explained by the need for healthcare workers with non-judgmental and non-stigmatising attitudes. A global systematic review and meta-analysis showed that decentralisation and integration of HCV care to harm reduction sites improved access to HCV testing, linkage to care and HCV treatment [37]. Similar results were already highlighted in several studies in different settings. For example, a knowledge synthesis on barriers and facilitators of HCV treatment in high income countries showed that trustworthy, understanding and culturally competent health workers were seen as facilitators to HCV treatment among priority populations such as people who inject drugs [38]. Recommendations from a HCV cascade analysis in Georgia among people participating in a methadone program advised the implementation of integrated harm reduction and treatment services, with strong adherence support provided by peers and professionals at treatment sites [39]. Also, a review concerning the response to HCV in Europe among people who inject drugs included community-based harm reduction and care services, as well as peer support, in the components of comprehensive HCV care services to which people who inject drugs should have access [40].

The need for a community-based/friendly approach is in accordance with the more global issue of access to care for culturally and linguistically diverse people [41, 42]. The development of cultural competence could fill the gap between specific needs with regard to access to care and the services provided for marginalised populations [43–47]. One of the most commonly used definitions of the cultural competence approach is “a set of congruent behaviours, attitudes, and policies that come together in a system, agency or among professionals and enable that system, agency or those professions to work effectively in cross-cultural situations” [48]. This definition emphasises that cultural competence should be implemented on different levels of care to address the multi-level barriers of health access.

Several studies confirm the need to reinforce and continue the efforts to provide HCV services in community-based harm reduction services to better address the needs of people who inject drugs and reduce stigma associated with drug injection. A study conducted in Myanmar showed the feasibility and the effectiveness of decentralised community-based HCV testing and treatment among people who inject drugs and people with liver disease in two sites [49]. Results showed that 86% of participants who were eligible (*n* = 489) to initiate HCV treatment (after a HCV testing) achieved sustained virological response at 12 weeks post treatment completion [49]. These results were similar among people who inject drugs and other participants. The site which targeted people who inject drugs had HRS such as needle and syringe distribution and was located near the major methadone treatment centre [49]. These data are in accordance with our study, which also highlighted the preferences for HCV testing and treatment decentralised in CBC or support centres for people who use drugs and confirmed that decentralised and integrated HCV services could be an effective way to reach people who inject drugs. Another study investigated the HCV treatment cascade integrated in a community-based HRS for people who inject drugs in Australia and showed an increase in the retention at each step of the cascade of care compared to data published before the DAAs introduction [50]. Results of Morris et al. [50] were also slightly better than in a study conducted after introduction of DAAs but which was based on nurse-led model [51]. This study indicated that 62% of participants started the treatment and 54% obtained a sustained virological response [51] compared to 72% and 44%, respectively [50]. However, Harney et al. [51] study had a very small sample (*n* = 24).

Future studies and policy guidelines on HCV services should take into account the specific needs of people who inject drugs. Diversifying HCV services offered by a community-based integration of HCV services could lead to an improvement of the HCV continuum of care and impede HCV transmission.

This analysis had some limitations. Results of this survey cannot be generalised to all of the communities of people who inject drugs due to the sampling method and small sample size. Specifically, almost half of people who/have inject(ed) drugs in our sample had already been treated for HCV. Therefore, barriers for HCV services could differ among people who inject drugs who had not yet been treated. The small sample size of this study also did not allow to comparison values and preferences of people who inject drugs who had already been tested and treated for HCV with those who did not.

Respondents who currently or formerly inject(ed) drugs were identified based on self-identification with these groups and not on recent or past injection behaviours. Furthermore, online surveys may not reach populations that do not have regular internet access such as those who are homeless or experience marginalisation, issues which can frequently occur among people who inject drugs. Finally, the survey was only available in English. Additional work is needed to collect information regarding values and preferences among marginalised populations who are most likely not reached by traditional health systems and are likely to encounter barriers to health services.

The main policy implications of these survey findings was to complement the strong evidence base from a global systematic review for simplified service delivery [33]. This led to a strong recommendation in the WHO guidelines for delivery of fully decentralised HCV testing and treatment and integration with existing services at harm reduction sites, and task sharing of activities to nurses and non-specialist doctors [52]. The impact on access of a decentralised, co-located “one-stop shop” for HCV testing and treatment will be greatest among people who inject drugs, who have particular difficulties accessing health services and have high rates of loss to follow-up. Delivery of HCV testing, care and treatment ideally as a “one-stop-shop” physically closer to the patient will also be more convenient, with fewer visits and potentially lower transport costs and less time off work. This study also indicated the need to move toward a tailored delivery of HCV testing and treatment which also considers drug injection-associated stigma. This could be developed with a strong implication of peer workers in the HCV services and through the integration of HCV services in HCV community-based settings.

## Conclusions

People who currently/formerly inject drugs showed specific needs concerning HCV services. Integration of HCV services in community-based harm reduction services is an important element for the development of adapted services to increase HCV care in this population and move toward eliminating the HCV.

## Data Availability

All data supporting the results of the present analysis are included in the manuscript.
